# Soil Microbiomes With the Genetic Capacity for Atmospheric Chemosynthesis Are Widespread Across the Poles and Are Associated With Moisture, Carbon, and Nitrogen Limitation

**DOI:** 10.3389/fmicb.2020.01936

**Published:** 2020-08-12

**Authors:** Angelique E. Ray, Eden Zhang, Aleks Terauds, Mukan Ji, Weidong Kong, Belinda C. Ferrari

**Affiliations:** ^1^School of Biotechnology and Biomolecular Sciences, University of New South Wales, Sydney, NSW, Australia; ^2^Australian Antarctic Division, Department of Environment, Antarctic Conservation and Management, Kingston, TAS, Australia; ^3^Key Laboratory of Alpine Ecology and Biodiversity, Institute of Tibetan Plateau Research, Chinese Academy of Sciences, Beijing, China

**Keywords:** carbon fixation, atmospheric chemosynthesis, trace gases, photosynthesis, environmental drivers, quantitative PCR

## Abstract

Soil microbiomes within oligotrophic cold deserts are extraordinarily diverse. Increasingly, oligotrophic sites with low levels of phototrophic primary producers are reported, leading researchers to question their carbon and energy sources. A novel microbial carbon fixation process termed atmospheric chemosynthesis recently filled this gap as it was shown to be supporting primary production at two Eastern Antarctic deserts. Atmospheric chemosynthesis uses energy liberated from the oxidation of atmospheric hydrogen to drive the Calvin-Benson-Bassham (CBB) cycle through a new chemotrophic form of ribulose-1,5-bisphosphate carboxylase/oxygenase (RuBisCO), designated IE. Here, we propose that the genetic determinants of this process; RuBisCO type IE (*rbcL1E*) and high affinity group 1h-[NiFe]-hydrogenase (*hhyL*) are widespread across cold desert soils and that this process is linked to dry and nutrient-poor environments. We used quantitative PCR (qPCR) to quantify these genes in 122 soil microbiomes across the three poles; spanning the Tibetan Plateau, 10 Antarctic and three high Arctic sites. Both genes were ubiquitous, being present at variable abundances in all 122 soils examined (*rbcL1E*, 6.25 × 10^3^–1.66 × 10^9^ copies/g soil; *hhyL*, 6.84 × 10^3^–5.07 × 10^8^ copies/g soil). For the Antarctic and Arctic sites, random forest and correlation analysis against 26 measured soil physicochemical parameters revealed that *rbcL1E* and *hhyL* genes were associated with lower soil moisture, carbon and nitrogen content. While further studies are required to quantify the rates of trace gas carbon fixation and the organisms involved, we highlight the global potential of desert soil microbiomes to be supported by this new minimalistic mode of carbon fixation, particularly throughout dry oligotrophic environments, which encompass more than 35% of the Earth’s surface.

## Introduction

Dry oligotrophic environments encompass more than 35% of the Earth’s surface (Mares and History, 1999). Due to global warming, dry environments are expected to expand to cover up to 56% of the Earth’s surface by the end of the 21st century (Cherlet et al., 2018). Despite their exposure to frequent freeze-thaw cycles, intense UV radiation, and limited carbon, nitrogen, and moisture availability (Wynn-Williams, 1990; Yergeau et al., 2006; Margesin and Miteva, 2011; Pearce, 2012; Cowan et al., 2014), polar soil microbiomes are diverse and abundant, driving important ecological processes (Yergeau et al., 2006; Cowan et al., 2014; Kleinteich et al., 2017). Such cold desert soil microbiomes are often dominated by *Actinobacteria*, *Proteobacteria*, and *Bacteroidetes* (Tindall, 2004; Cary et al., 2010; Bottos et al., 2014; Tytgat et al., 2014; Zhang et al., 2020), with a high abundance of phototrophic primary producers, particularly *Cyanobacteria* and algae (Friedmann, 1982; Elster, 2002; Namsaraev et al., 2010; Jansson and Taş, 2014). However, in ice-free polar deserts, these phototrophs are often restricted to lithic niches that protect them from intense UV radiation and desiccation (Walker and Pace, 2007; Pointing et al., 2009; Wierzchos et al., 2012; Cowan et al., 2014; McKay, 2016; Goordial et al., 2017). Oligotrophic deserts comprising little to no detectable photoautotrophs are distributed worldwide, leading researchers to question what carbon and energy sources support the microbial communities functioning in these harsh ecosystems (Warren-Rhodes et al., 2006; Albertsen et al., 2013; Ferrari et al., 2015; Ji et al., 2015; Tebo et al., 2015; Zhang et al., 2020).

In a recent study by Ji et al. (2017), a novel form of light-independent autotrophy termed atmospheric chemosynthesis was discovered in soils across two oligotrophic East Antarctic deserts; the arid Robinson Ridge (average organic carbon 0.17%, moisture 4.4%) in the Windmill Islands and the hyper-arid Adams Flat (average organic carbon 0.09%, moisture 0.42%) in the Vestfold Hills region. At these sites, H_2_-oxidizing bacteria were proposed to employ high-affinity type 1h-[NiFe]-hydrogenases to scavenge and oxidize hydrogen gas that has diffused into the subsurface soil from the atmosphere. The energy liberated from this oxidation process was proposed to support cell maintenance as well as carbon fixation *via* the Calvin-Benson-Bassham (CBB) cycle, and was linked to a novel chemotrophic form of ribulose-1,5-bisphosphate carboxylase/oxygenase (RuBisCO), type IE (Grostern and Alvarez-Cohen, 2013; Tebo et al., 2015; Ji et al., 2017). RuBisCO type IE (*rbcL1E*) is phylogenetically distinct from the photoautotrophic RuBisCO types IA and IB and is notably also distinct from the other chemoautotrophic RuBisCO red-types IC and ID, diverging from these clades prior to their own separation (Park et al., 2009; Tebo et al., 2015). Despite this discovery, the broader ecological role and significance of this novel RuBisCO has still not been determined.

Here, we propose that terrestrial microbiomes inhabiting cold oligotrophic deserts throughout the world may be genetically capable of supporting cell growth through atmospheric chemosynthesis, particularly in environments where photoautotrophs are limited. We used quantitative PCR (qPCR) targeting the *rbcL1E* and the 1h-[NiFe]-hydrogenase large subunit (*hhyL*) genes to survey 122 desert soils spanning the Tibetan Plateau and 13 Antarctic and high Arctic sites. The taxonomic composition of each soil was analyzed using amplicon sequencing. We also aimed to identify the abiotic parameters associated with the genetic capacity for atmospheric chemosynthesis within each region, by correlating the relative abundances of *rbcL1E* and *hhyL* against 26 measured soil physicochemical parameters. We hypothesize that atmospheric chemosynthesis, as a new form of chemoautotrophy, is associated with low moisture and nutrient limitation in cold desert soils, under the general exclusion of phototrophs.

## Materials and Methods

### Site Descriptions

**Figure 1:**
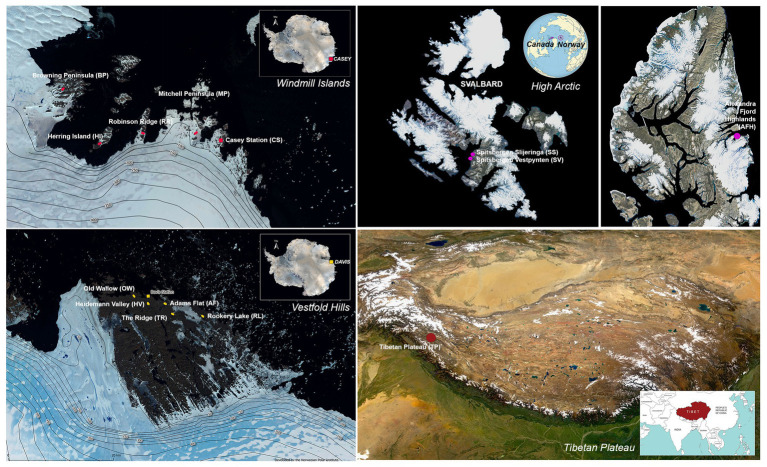
Map of the 14 sites studied with approximate sampling locations across the Vestfold Hills and Windmill Island regions of Eastern Antarctica, as well as the high Arctic and the Tibetan Plateau. Satellite image (Quantarctica v3 GIS package in QGIS 3.4.7).

Sampling was conducted across 14 cold desert sites spanning Antarctica (the Windmill Islands and Vestfold Hills), the high Arctic and the Tibetan Plateau (Figure 1). The Windmill Islands is an ice-free region located in Wilkes land, Eastern Antarctica (Gasparon et al., 2007). Centered at 110°30′E and 66°22′S, the region covers an area of 75 km^2^ (Goodwin, 1993), has an elevation of below 100 m and includes five major peninsulas and multiple rocky islands (Siciliano et al., 2014; Bissett et al., 2016). Five sites from the Windmill Islands were sampled in this study; Mitchell Peninsula (MP; 66°31′S, 110°59′E), Robinson Ridge (RR; 66°22′S, 110°35′E), Browning Peninsula (BP; 66°27′S, 110°32′E), Herring Island (HI; 66°24′S, 110°39′E), and Casey station (CST; 66°16′S, 110°31′E).

Soil samples were also obtained from five sites near Davis station (68°35′S, 77°58′E), the most southerly research station in Eastern Antarctica; Old Wallow (OW; 68°36’S, 77°57′E), Heidemann Valley (HV; 68°35’S, 78°0′E), Adams Flat (AF; 68°33’S, 78°1′E), Rookery Lake (RL; 68°30’S, 78°7′E), and The Ridge (TR; 68°54′S, 78°07′E). These sites are in the low-lying Vestfold Hills region of Eastern Antarctica, a region consisting of numerous deep-sea inlets and lakes (Zhang et al., 2020). High Arctic surface soils were previously collected from a Canadian site, Alexandra Fjord Highlands (AFH; 78°51′N, 75°54′W), and two Norwegian sites; Spitsbergen Slijeringa (SS; 78°14′N, 15°30′W) and Spitsbergen Vestpynten (SV; 78°14′N, 15°20′W). Soil samples were also collected from the cold, high-altitude Qinghai-Tibet Plateau in Western China (TP; 32°27′N, 80°4′E), which has been previously referred to as Earth’s third pole (Gao et al., 2018).

### Soil Sampling

Soil samples were obtained from the 13 high Arctic and Antarctic sites by Australian Antarctic Program (AAP) expeditioners, while the Tibetan Plateau soil samples were obtained by expeditioners from the Chinese Academy of Sciences. In total, 122 samples were collected from the top 10 cm of soil, as previously described (Siciliano et al., 2014; Ji et al., 2020; Zhang et al., 2020). All soil samples were stored at −80°C until downstream analysis.

### Soil Physicochemical Analysis

For all the Antarctic and high Arctic soil samples (*n* = 117), physicochemical parameters (*n* = 26) were quantified using standard procedures described previously by Siciliano et al. (2014) and van Dorst et al. (2014b) (Supplementary Materials 1–3). Briefly, chemical parameters included those obtained by X-Ray fluorescence elemental analysis (SiO_2_, TiO_2_, Al_2_O_3_, Fe_2_O_3_, MnO, MgO, CaO, Na_2_O, K_2_O, and P_2_O_5_) and water extraction (Cl^−^, Br^−^, NO_3_^−^, NO_2_^−^, PO_4_^3−^, and SO_4_^2−^). Laser scatter was used to quantify soil particle size, while pH and conductivity were measured using a 1:5 soil to distilled water suspension. Global positioning system (GPS), geographic information system (GIS), and digital elevation models (DEMs) were implemented to measure physical parameters, including location, elevation, and aspect. Combustion and nondispersive infrared (NDIR) gas analysis were used to quantify total carbon (TC), total phosphorus (TP), and total nitrogen (TN) content (Rayment and Lyons, 2011). As comprehensive soil physicochemical analysis was not performed on the Tibetan Plateau soils, these samples were excluded from downstream correlation analysis.

### Community Genomic DNA (gDNA) Extraction

DNA was extracted in triplicate from 0.25 to 0.3 g of each soil sample using the FastDNA SPIN kit for soil (MP Biomedicals, NSW, Australia) as per the manufacturer’s instructions. DNA was quantified with Picogreen (Life Technologies, Vic, Australia) and fluorescence measured on a fluorescence plate reader (SpectraMax M3 Multi-Mode Microplate Reader; Molecular Devices, CA), prior to being stored at −80°C until used.

### Bacterial 16S rRNA Gene Sequencing and Data Analysis

Barcode tag amplification of the bacterial 16S ribosomal RNA (rRNA) gene was previously performed on soil gDNA using primers 28F and 519R (Bissett et al., 2016; Ji et al., 2020; Zhang et al., 2020). ARISA analysis confirmed that each set of triplicate gDNA extractions were significantly correlated (data not shown; van Dorst et al., 2014b; Ferrari et al., 2015). Therefore, 16S rRNA sequencing and all downstream analysis was performed using a single gDNA extract from each of the soil samples. Paired-end amplicon sequencing was performed using the Illumina MiSeq platform (Illumina, 312 California, US) in accordance with protocols from the Biome of Australia Soil Environments (BASE) project by Bioplatforms Australia (Bissett et al., 2016). The Antarctic and high Arctic data were downloaded together from the Australian Antarctic Datacentre^1^, and the BASE repository^2^. Amplicon sequencing data for the Tibetan Plateau soils was obtained from Ji et al. (2020) and analyzed separately. Open operational taxonomic unit (OTU) picking, assignment and classification were performed according to previously described methods (Zhang et al., 2020). In brief, USEARCH v10.0.240 (Edgar et al., 2011) and VSEARCH v2.8.0 (Torbjørn et al., 2016) were employed according to the UPARSE-OTU algorithm (Edgar, 2013). Sequences were quality filtered, trimmed, and clustered *de novo* to classify OTUs at 97% identity, and assigned to separate sample-by-OTU matrices where singletons were discarded manually. Sequences were then taxonomically classified against the SILVA v3.2.1323 SSU rRNA database (Quast et al., 2013).

### Validation of the RuBisCO Type IE qPCR Primer Set

The RuBisCO type IE qPCR primer set (rbcL1Ef/rbcL1Er) designed in Ji et al. (2017) was validated for use in polar soils by amplicon sequencing DNA lysates. PCR was performed in reaction mixtures composed of 2 μl template gDNA, 5 μl GoTaq Flexi Buffer; pH 8.5 (Promega Corporation, USA), 1 μl 25 mM MgCl2, 0.5 μl 10 mM dNTPs (Bioline), 13.75 μl UltraPure DNase/RNase-free distilled water (Invitrogen, Scotland), 0.312 μl of each 40 μM primer (rbcL1Ef/rbcL1Er; Table 1; Integrated DNA Technologies), 0.126 μl GoTaq polymerase (Promega Corporation, USA), and 2 μl of 1 mg/μl Bovine Serum Albumin (BSA). Amplifications were conducted using a Mastercycler nexus X2 (Eppendorf, NSW, Australia) under the following conditions; 95°C for 5 min, 35 cycles of denaturing at 95°C for 30 s, annealing at 55°C for 30 s and extension at 72°C for 30 s, and a final extension of 72°C for 5 min. PCR products underwent amplicon sequencing using the Illumina platform at the Australian Centre for Ecogenomics (University of Queensland). Sequences that lacked both the forward and reverse primer binding sites were discarded, as were those with average quality scores <35. The remaining sequences were matched to reference RuBisCO subtype sequences using the National Centre for Biotechnology Information (NCBI) Basic Local Alignment Search Tool (BLAST; Ye et al., 2012).

**Table 1:** Quantitative PCR (qPCR) primer sets used in this study.

Target gene	Primer name and sequence (5'-3')		Target size (bp)	Reference
	Forward	Reverse		
1h-[NiFe]-hydrogenase large subunit gene (*hhyL*)	NiFe-244f; GGGATCTGCGGGGACAACCA	NiFe-568r; TCTCCCGGGTGTAGCGGCTC	324	(Constant et al., 2010)
RuBisCO type IE (*rbcL1E*)	rbcL1Ef; GGACBGTSGTVTGGACSGA	rbcL1Er; TTGAABCCRAAVACRTTGC	187	(Ji et al., 2017)
16S rRNA gene	Eub1048f; GTGSTGCAYGGYTGTCGTCA	Eub1194r; ACGTCRTCCMCACCTTCCTC	146	(Maeda et al., 2003)

### qPCR Analysis of *rbcL1E* and *hhyL* Genes

qPCR was performed on all 122 gDNA extracts using previously published qPCR primers targeting *rbcL1E* (rbcL1Ef/rbcL1Er; Ji et al., 2017), *hhyL* (NiFe-244f/NiFe-568r; Constant et al., 2010, 2011), and the 16S rRNA gene (Eub1048f/Eub1194r; Maeda et al., 2003; Table 1). Positive controls were synthetically designed gene fragments (gBlocks; Integrated DNA Technologies, VIC, Australia) composed of representative *rbcL1E* (JX458468.1), *hhyL* (AB894417.1), and 16S rRNA (MF689012.1) gene sequences. Standard curves were generated over 5–7 orders of magnitude.

qPCR reaction mixtures for *rbcL1E* and the *hhyL* target genes were prepared using 10 μl QuantiFast SYBR Green PCR Master Mix (Qiagen, VIC, Australia), 0.5 μl of each 40 μM primer (Integrated DNA Technologies), 8 μl UltraPure DNase/RNase-free distilled water (Invitrogen), and 1 μl diluted (1:10) gDNA. The reaction mix for the 16S rRNA gene was identical, except that 7 μl molecular water and 1 μl of 5 ng/μl T4gene32 Protein (Sigma-Aldrich, NSW, Australia) were added to reduce non-specific amplification (Baugh et al., 2001; Villalva et al., 2001). Thermocycling reactions was completed using a CFX96 Touch™ Real-Time PCR Detection System (Bio-Rad Laboratories, NSW, Australia) under standard two-step conditions; 94°C for 5 min, 45 cycles of 94°C for 20 s, and 54°C for 50 s, followed by a melt-curve step from 50 to 95°C. The quantitative fluorescence data were spectrophotometrically collected during the 54°C step.

### qPCR Data Analysis

CFX manager software (Bio-Rad Laboratories) was used for data analysis. Melt peak analysis confirmed amplification specificity. Amplification within the most dilute standard was detected at least 5 C_t_ before the negative template controls. The average C_t_ values across replicates were determined, and then standard curve efficiencies and copy numbers were converted into copies/g of soil. Here, the average efficiencies for the 16S rRNA, *rbcL1E* and *hhyL* genes qPCR reactions were 86.9% (±3.55), 91.1% (±1.48), and 89.9% (±3.06), respectively. The *R*^2^ value for each qPCR was equal to or greater than 0.99. In accordance with Jones and Hallin (2019), the genetic copy numbers of *rbcL1E* quantified were corrected against the proportion of *rbcL1E* target reads observed during site-specific primer validation [section “Validation of the RuBisCO Type IE qPCR Primer Set”]. Next, *rbcL1E* and *hhyL* copy numbers normalized against 16S rRNA gene copy numbers and expressed as a percentage. Beanplots comparing the relative abundances of *rbcL1E* and the *hhyL* gene were produced using BoxPlotR (Kampstra, 2008; RStudio and Inc, 2013; R Core Team, 2018).

### Multivariate Data Analysis

Skewness was eliminated from the 26 measured physicochemical factors using log or square root transformations within the PRIMER v7 + PERMANOVA package (Clarke and Warwick, 2001), and normalized (mean/standard deviation; van Dorst et al., 2014a). Subsequent multivariate data analysis of the physicochemical, 16S rRNA amplicon and qPCR data was carried out in the R v3.5.1 environment (R Core Team, 2018). Non-metric multi-dimensional scaling (NMDS) ordination plots were generated using the R package “vegan” (Oksanen et al., 2015) to visualize the ordering of samples in reduced two-dimensional space. Euclidean dissimilarity index was applied to the physicochemical parameters, while the Bray–Curtis dissimilarity index was applied to the bacterial taxonomic abundance dataset. Ellipse paths were calculated using the “veganCovEllipse” function developed by Torondel et al. (2016). The veg.dist() and anosim() function from the “vegan” R package was used to conduct one-way analysis of similarity (ANOSIM) on dissimilarity matrices on our OTU and physicochemical datasets, grouped on a regional and site level (permutations = 999, *α* = 0.05). Subsequently, the qPCR data was analyzed against the 26 measured physicochemical factors. To identify the most appropriate correlation method to apply to this analysis, the linearity between the genetic abundance of *rbcL1E* and *hhyL* with each physicochemical parameter was tested through the generation of scatterplots using the “ggscatter” function from the “ggplot2” R package (Wickham, 2016). Spearman correlations between *rbcL1E* and *hhyL* genetic abundances and each physicochemical condition were displayed using the R package “corrplot” (Wei et al., 2017) to determine the direction of correlations observed. Multivariate random forest regression analysis was subsequently conducted using the “rfPermute” package (Archer, 2013) under the R-environment^3^. The relative importance and the significance of environmental factors in explaining the relative abundance of *rbcL1E*, *hhyL*, and the total bacterial abundance were determined.

## Results

### Validation of the *rbcL1E* qPCR Primer Set

Following quality control, amplicon sequencing using the *rbcL1E* primer set resulted in a total of 760, 855 sequence reads. In these polar soils, the primers were on average 73.8% specific toward *rbcL1E* (Supplementary Material 4). The specificity of the *rbcL1E* primer pair ranged between 61.5 and 86.9% with most non-target sequences retrieved classified as RuBisCO type 1C. Genetic copies of *rbcL1E* obtained during later qPCR analysis were corrected against the site-specific primer specificities obtained here.

### Relative Abundances of *rbcL1E* and *hhyL* Genes in Cold Desert Soils

**Figure 2:**
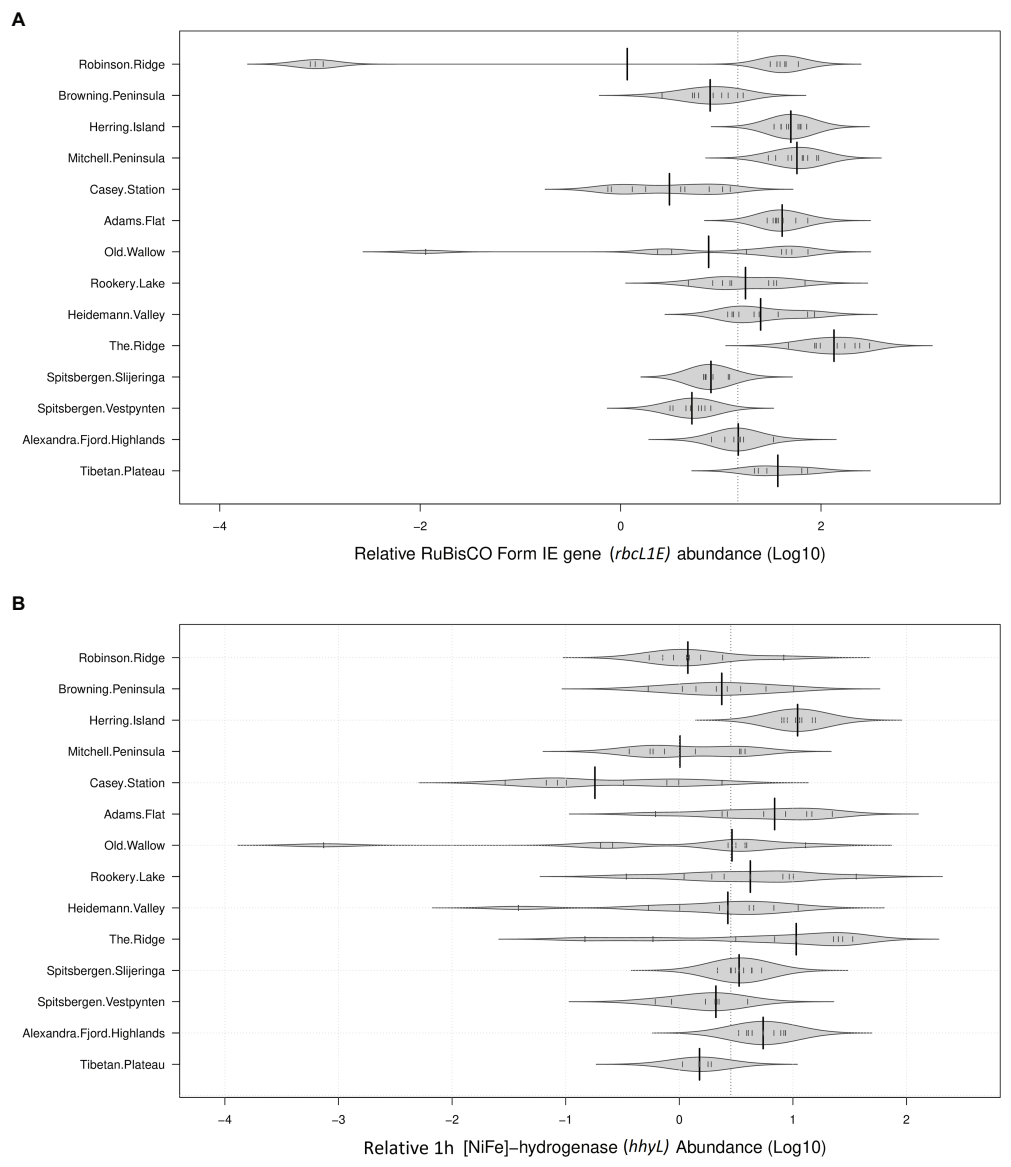
Relative abundances of target genes associated with atmospheric chemosynthesis within polar desert soils. The proposed genetic determinants of this process were widely distributed across all 14 sites spanning Antarctica, the high Arctic, and Tibetan Plateau **(A)**. Ribulose-1,5-bisphosphate carboxylase/oxygenase (RuBisCO) type IE (*rbcL1E*; **B**). 1h-[NiFe]-hydrogenase large subunit gene (*hhyL*). Large solid black lines indicate average relative abundances per site; the dotted black line indicates the mean relative abundance of all 14 sites (36.68% for *rbcL1E* and 5.22% for *hhyL*); the small, solid black lines represent individual data points; polygons represent the estimated density of the data.

The genetic determinants for atmospheric chemosynthesis were detected in high abundances across all 122 polar soils analyzed (Figure 2). As a percentage of 16S rRNA gene copies/g soil, average relative abundances of *rbcL1E* were highest within the Vestfold Hills (58.1%), followed by the Tibetan Plateau (42.1%), the Windmill Islands (31.0%), and the high Arctic (10.0%). The relative abundances of the *hhyL* gene were also variable, with the highest relative abundance observed in the Vestfold Hills (7.73%), followed by the Windmill Islands (3.95%), the high Arctic (3.86%), and Tibetan Plateau (1.21%).

The average copy numbers of *rbcL1E* and *hhyL* genes per site were highest within the Vestfold Hills at 3.42 × 10^8^ and 6.05 × 10^7^ copies/g soil, respectively. Within the Vestfold Hills, Adams Flat exhibited the highest relative abundance of both target genes (*rbcL1E* 7.24 × 10^8^ and *hhyL* 1.33 × 10^8^), while the lowest abundances were found at The Ridge (*rbcL1E* 5.68 × 10^7^ and *hhyL* 4.61 × 10^6^). In comparison to the Vestfold Hills, the relative abundances of *rbcL1E* and *hhyL* were lower on average within the Windmill Islands with 1.11 × 10^8^ and 1.20 × 10^7^ copies, respectively. Within the Windmill Islands, the *rbcL1E* gene abundances ranged between 2.32 × 10^7^ copies/g soil at Browning Peninsula and 2.72 × 10^8^ copies/g soil at Mitchell Peninsula. The lowest *hhyL* gene abundances in the Windmill Islands were observed for soils from the human impacted Casey station (4.18 × 10^6^ copies/g soil), with the highest numbers detected at Herring Island (3.39 × 10^7^ copies/g soil).

### Bacterial Communities Across the Poles

**Figure 3:**
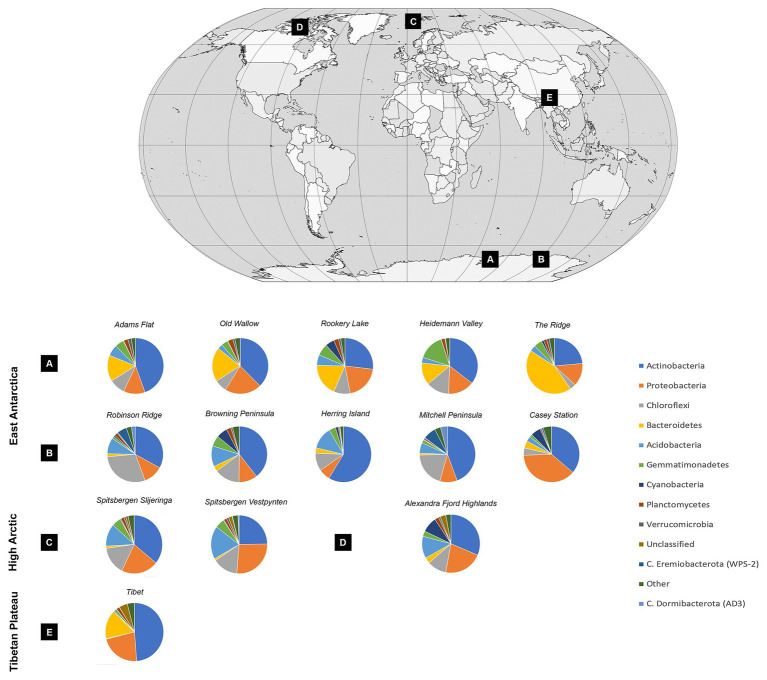
Soil bacterial community diversity at a phylum level across all 14 cold desert sites, and their locations throughout the **(A)** Vestfold Hills, **(B)** Windmill Islands, **(C)** Norway, **(D)** Canada, and **(E)** Tibetan Plateau. *Actinobacteria* dominated all soils, with average site-level relative abundances ranging from 23.6% at The Ridge to 58.6% at Herring Island. *Proteobacteria*, *Chloroflexi*, *Bacteroidetes*, and *Acidobacteria* were also highly abundant. In contrast, *Cyanobacteria* were present in low relative abundances accounting for <1% of the bacterial communities in eight sites, increasing to 5–9% within the Alexandra Fjord Highlands, Rookery Lake, Casey station, and Browning Peninsula. Within the Windmill Islands region, *Candidatus Eremiobacterota* was present in high relative abundances, with site-level averages ranging between 0.02% at Herring Island and 7.42% at Mitchell Peninsula. Similarly, *Candidatus Dormibacterota* was also present in high relative abundances in the Windmill Islands only.

Soil bacterial communities were dominated by *Actinobacteria* at most sites, with relative abundances ranging from 23.57% at the Ridge to 58.63% at Herring Island (Figure 3, Supplementary Material 5). While *Proteobacteria*, *Chloroflexi*, *Bacteroidetes*, and *Acidobacteria* were also highly abundant, *Cyanobacteria* were present in low relative abundances (<1%) at 8 of the 13 sites (Spitsbergen Vestpynten, Spitsbergen Slijeringa, Heidemann Valley, Old Wallow, Adams Flat, Mitchell Peninsula, Robinson Ridge, and the Tibetan Plateau). In contrast, the relative abundances of *Cyanobacteria* were higher at 5–9% within the Alexandra Fjord Highlands, Rookery Lake, Casey station, and Browning Peninsula. Candidate phyla accounted for substantial proportions of the bacterial communities particularly within the Windmill Islands, dominating microbial communities at Mitchell Peninsula (12.71%), Robinson Ridge (8.71%), and Casey station (4.52%). Of the candidate phyla residing in the Windmill Islands, *Candidatus Eremiobacterota* (WPS-2) was the most abundant comprising 7.42% relative abundances on average at Mitchell Peninsula, followed by *Candidatus Dormibacterota* (AD3) at an average relative abundance of 4.06% at Mitchell Peninsula.

### Soil Physicochemistry and Bacterial Community Similarity Across East Antarctica and the High Arctic

**Figure 4:**
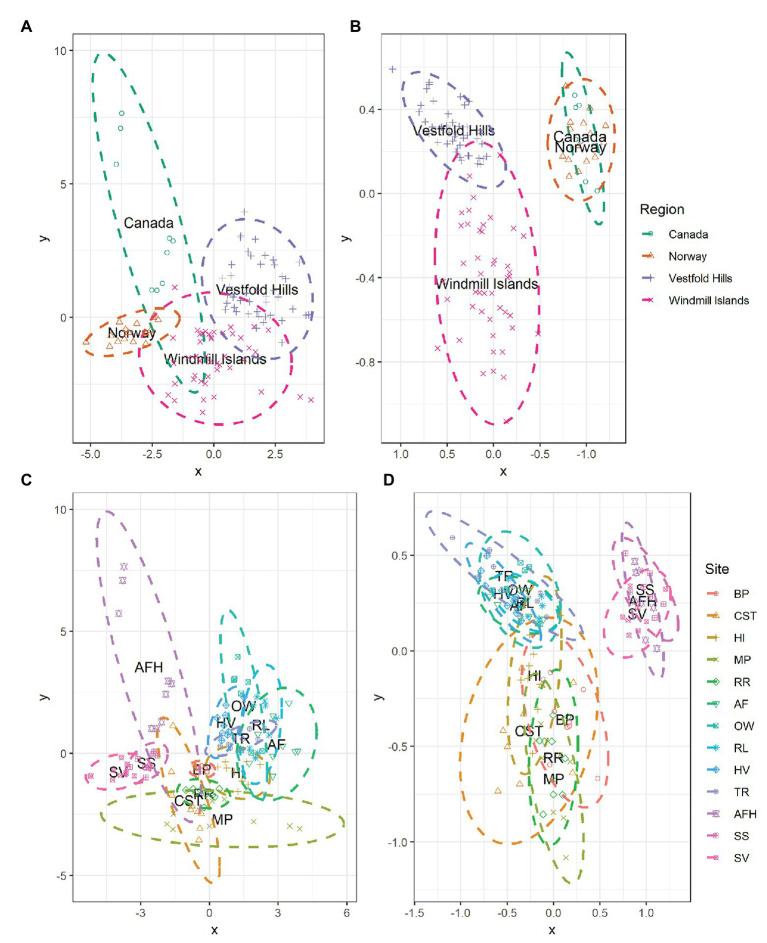
NMDS plots showing the relationships among samples on a regional and local scale for **(A,C)** measured soil physicochemical parameters and **(B,D)** bacterial community composition at phylum level. Soil samples displayed greater environmental and bacterial community similarities within rather than between regions, as indicated by the formation of regional-level clustering. Bacterial communities within the high Arctic samples (Norway and Canada) were highly similar to each other with the clusters overlapping. Soil samples clustered according to site, indicating that soils from the same location share similar environmental conditions and bacterial community structures. For both environmental and bacterial communities, the greatest dissimilarity was observed within the Windmill Islands although variation in physicochemical parameters was also observed across the high Arctic sites, predominantly Alexandra Fjord Highlands.

At the global scale, the bacterial communities were more similar within, rather than between regions as indicated by the distinct formation of regional clusters when viewed using an NMDS plot (Figure 4B). Accordingly, ANOSIM results indicated that bacterial community composition varied significantly between the Antarctic and the high Arctic samples (ANOSIM *R* = 0.520, *p* = 0.001), with significant differences also observed on regional level between Norway, Canada, the Windmill Islands, and Vestfold Hills (ANOSIM *R* = 0.872, *p* = 0.001). Site-level bacterial community similarities have also been visualized, with the greatest variations occurring within Mitchell Peninsula and Alexandra Fjord Highlands (Figure 4D). Bacterial communities within soils from the three high Arctic sites were significantly similar to each other (ANOSIM *R* = 0.328, *p* = 0.002), as were soils sampled from within the Vestfold Hills (ANOSIM *R* = 0.276, *p* = 0.001). Comparatively, a significant and more substantial variation in bacterial composition was observed within the Windmill Island sites (ANOSIM *R* = 0.476, *p* = 0.001).

The measured soil physicochemical properties mirrored the bacterial community data, with distinct regional clusters showing that soils were more similar within than between regions (Figure 4A). The associated ANOSIM results indicated that physicochemical conditions varied significantly between the Antarctic and the high Arctic samples (ANOSIM *R* = 0.626, *p* = 0.001), with differences also observed on a regional level between Norway, Canada, the Windmill Islands, and Vestfold Hills (ANOSIM *R* = 0.730, *p* = 0.001). Site-level variations in physicochemical conditions have also been visualized (Figure 4C). On this more localized level, soil samples from within the Vestfold Hills demonstrated a high degree of physicochemical similarity to each other compared to those from other regions (ANOSIM *R* = 0.302, *p* = 0.001). High Arctic soils were also more physicochemically like each other than soils from other regions (ANOSIM *R* = 0.302, *p* = 0.001). In contrast, the Windmill Island soil samples demonstrated greater dissimilarity within sites (ANOSIM *R* = 0.617, *p* = 0.001).

The East Antarctic soils (*n* = 90) analyzed in this study were low in moisture (0.0023–0.26%) and nutrients, particularly TC (0.008–2.58%), TN (0.0065–0.22%), and TP (0.027–0.23%; Supplementary Materials 1, 2). Within the high Arctic (*n* = 27), these values were higher (TN was 0.022–0.080% in Canada and 0.073–0.43% in Norway; TC was 0.29–6.89% in Canada and 1.15–6.55% in Norway; Moisture was 0.042–0.11% in Canada and 0.11–0.42% in Norway; Supplementary Material 3). Soils obtained from the Windmill Islands and Norway were acidic (average pH 6.08 and 5.97, respectively), while soils from the Vestfold Hills and Canada were more alkaline (average pH 8.49 and 7.81, respectively; Supplementary Materials 1–3).

**Figure 5:**
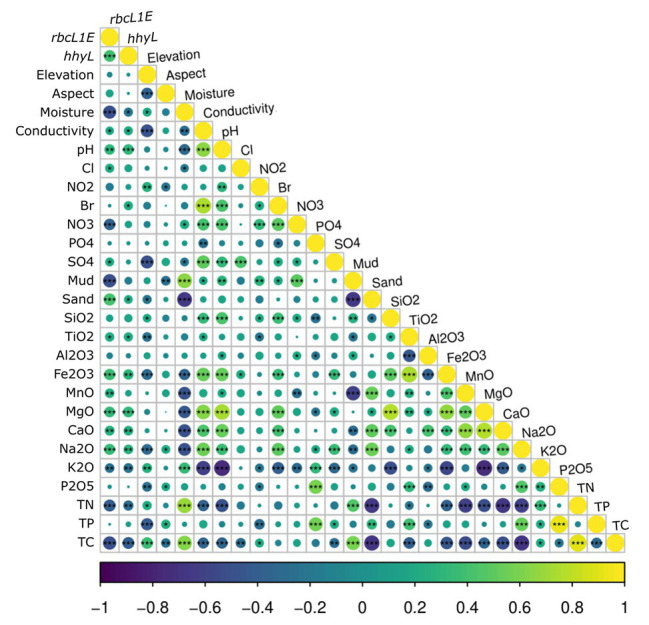
Spearman correlations between the relative abundance of the two target genes, *rbcL1E* and *hhyL*, against all 26 measured physicochemical parameters for the 117 Antarctic and high Arctic soils. *rbcL1E* and *hhyL* each produced positive correlations with soil conductivity, pH, and sand composition as well as with greater abundances of various oxides. *rbcL1E* and *hhyL* both occurred in higher abundance within samples of low moisture, total carbon (TC), and total nitrogen (TN). *rbcL1E* also occurred in higher abundance within samples of low NO_3_^−^ and mud composition.

Multivariate random forest analysis revealed strong and significant relationships between the genetic abundances of *rbcL1E*, *hhyL*, and multiple environmental parameters, while Spearman correlations showed the direction of these relationships. The relative abundances of *rbcL1E* and *hhyL* were most significantly explained by soil moisture (IncMSE = 69.35 and 24.38, respectively), TC (IncMSE = 16.45 and 17.48, respectively), and mud composition (IncMSE = 28.73 and 15.61, respectively), with significantly greater (*p* < 0.05; Table 2) abundances of both genes occurring under low moisture, carbon and mud composition (Figure 5). Additionally, *rbcL1E* was significantly explained by various oxides including NO_3_ (IncMSE = 32.83), Na_2_O (IncMSE = 24.40), MgO (IncMSE = 19.47), CaO (IncMSE = 26.44), and MnO (IncMSE = 16.09; *p* < 0.05; Table 2). Spearman analysis revealed that in these cases, greater *rbcL1E* was associated with lower NO_3_ content and greater levels of the other oxides (Figure 5). Random forest analysis revealed that multiple environmental factors significantly influenced microbial community structure, including pH (IncMSE = 31.35), conductivity (IncMSE = 23.86), TN (IncMSE = 26.91), K_2_O (IncMSE = 27.04), Al_2_O_3_ (IncMSE = 20.59), SiO_2_ (IncMSE = 16.87), PO_4_ (IncMSE = 17.23), and NO_2_ (IncMSE = 12.83; *p* < 0.05), however, these factors did not significantly explain either genetic determinant of atmospheric chemosynthesis (Table 2).

**Table 2:** Random forest analysis of the genetic abundances of *hhyL* and *rbcL1E*, relative to 16S rRNA genes, and 16S rRNA genes against 26 physicochemical parameters.

	*hyyL*/16S rRNA		*rbcL1E*/16S rRNA		16S rRNA	
	%IncMSE	%IncMSE.pval	%IncMSE	%IncMSE.pval	%IncMSE	%IncMSE.pval
Moisture	24.38	**0.002**	69.35	**0.001**	26.19	**0.003**
Mud	15.61	**0.034**	28.73	**0.003**	40.45	**0.001**
TC	17.48	**0.019**	16.45	**0.023**	27.47	**0.004**
pH	11.64	0.077	14.7	0.052	31.35	**0.002**
Elevation	13.12	**0.046**	9.49	0.105	31.81	**0.001**
NO_3_	6	0.266	32.83	**0.002**	15.28	**0.033**
Na_2_O	−2.71	0.981	24.4	**0.004**	17.63	**0.026**
MgO	10.71	0.135	19.47	**0.013**	21.33	**0.008**
CaO	10.87	0.130	26.44	**0.005**	11.92	0.139
TN	7.48	0.323	11.84	0.075	26.91	**0.003**
Aspect	3.96	0.421	18.61	**0.025**	19.25	**0.028**
K_2_O	5.6	0.459	8.55	0.145	27.04	**0.001**
MnO	6.39	0.392	16.09	**0.037**	17.99	**0.033**
Al_2_O_3_	7.65	0.218	11.33	0.070	20.59	**0.013**
Cl	15.37	**0.031**	14.5	**0.045**	8.84	0.199
Conductivity	8.61	0.218	5.74	0.282	23.86	**0.006**
Sand	2.67	0.686	22.82	**0.009**	9.06	0.261
Fe_2_O_3_	4.04	0.690	12.49	0.080	12.94	0.121
SiO_2_	4.82	0.601	6.94	0.251	16.87	**0.037**
TiO_2_	6.35	0.375	8.32	0.170	13.63	0.078
PO_4_	5.06	0.327	5.88	0.215	17.23	**0.025**
NO_2_	4.44	0.182	8.29	0.087	12.83	**0.040**
SO_4_	7.78	0.202	7.18	0.202	10.09	0.175
P_2_O_5_	5.58	0.472	7.56	0.221	10.59	0.186
Br	3.62	0.411	3.71	0.337	15.53	**0.017**
TP	2.49	0.724	4.11	0.398	12.79	0.089

Bold text = significant *p* values (<0.05).

## Discussion

Atmospheric hydrogen oxidation has only recently been identified as an energetic driver of microbial autotrophic CO_2_ fixation through the CBB cycle (Ji et al., 2017). Until now, atmospheric chemosynthesis has been overlooked as a niche process with unknown global significance. Here, we confirm that the genetic determinants of this new form of chemoautotrophy (*rbcL1E* and *hhyL*) are widespread and abundant throughout soil microbiomes of geographically distinct polar regions throughout Antarctica, the high Arctic, and the Tibetan Plateau. These findings support the hypothesis that this minimalistic carbon fixation strategy may be considered a globally occurring phenomenon and an important widespread survival adaptation in oligotrophic desert soil ecosystems.

While the role of hydrogen oxidation in contributing to microbial primary production is newly discovered, the role of high affinity hydrogenases (*hyyL*) in fulfilling the energy requirements of dormant soil bacteria is well established (Constant et al., 2008, 2010, 2011; Berney and Cook, 2010; Berney et al., 2014; Greening et al., 2015; Islam et al., 2019; Piche-Choquette and Constant, 2019). During periods of extreme environmental stress, H_2_-oxidizers can reversibly lower their metabolic activity and thereby, their energy requirements (Lennon and Jones, 2011). Under these conditions, the aerobic oxidation of atmospheric hydrogen provides bacteria with a ubiquitous and reliable source of energy (Morita, 1999; Smith-Downey et al., 2008; Constant et al., 2011). The process is indeed widespread, with *hyyL* reported at abundances of 10^6^–10^8^ genetic copies per gram of soil in both oligotrophic and copiotrophic ecosystems (Constant et al., 2011). Moreover, greater *hhyL* expression and hydrogen oxidizing activity have been linked to environments with lower organic carbon content (King, 2003; Greening et al., 2015), with H_2_-oxidizers reported to be among the earliest colonizers of volcanic deposits, despite the negligible amounts of organic matter present (King, 2003; Sato et al., 2004). Here, we also revealed the presence of high numbers of *hhyL* genes (4.18 × 10^6^–3.39 × 10^7^ copies/g soil) in cold desert soils from across the three poles, many of which contained extremely low levels of carbon and nitrogen. We note here that although the qPCR primer are widely implemented (Constant et al., 2010, 2011; Meredith et al., 2014; Khdhiri et al., 2015; Piché-Choquette et al., 2016), the discovery of high-affinity hydrogenases beyond group 1 h (D. Cowan, personal communication; Greening et al., 2014; Islam et al., 2020) suggests that the high-affinity hydrogenase gene abundances quantified here are underestimations.

Despite the widespread and abundant distribution of *hhyL*, the global co-occurrence of *hhyL* and *rbcL1E* has been unknown. The high and widespread co-occurrence of *hhyL* and *rbcL1E* across all 122 soils analyzed here indicates that the energy liberated from atmospheric hydrogen oxidation may be directed toward bacterial cell growth and primary production more pervasively than anticipated. Previous studies have indicated that trace gas chemosynthetic bacteria belong to the phyla *Actinobacteria*, *C. Eremiobacterota*, and *C. Dormibacterota* phyla (Park et al., 2009; Ji et al., 2017). The *rbcL1E* gene has also been detected within *Chloroflexota*, *Firmicutes*, and *Verrucomicrobiota* (Tebo et al., 2015). In this study, these taxa dominated soil communities from across the three poles, together accounting for up to 76.2% of the microbial community composition (Figure 3; Supplementary Material 5). Thus, in cold nutrient-starved deserts, trace gas chemoautotrophs appear to have a selective advantage for survival.

It has been proposed that atmospheric chemosynthesis and photosynthesis are both contributors to microbial primary production in oligotrophic environments, with contributions likely to vary along an aridity gradient (Ji et al., 2017; Bay et al., 2018). Indeed, variability in photo and chemoautotrophic potential was observed here with abundances of *rbcL1E* being particularly low in the high Arctic (10.0%), Casey station (4.8%), and Browning Peninsula (9.0%) soils (Figure 2). Each of these sites also contained greater photosynthetic potential than the other sites due to higher abundances of *Cyanobacteria* (5.7–8.6%; Kleinteich et al., 2017; Pudasaini et al., 2017; Zhang et al., 2020). *rbcL1E* gene abundances were also more variable than *hhyL*, reflecting the more widespread role of the high affinity hydrogenases in supplying maintenance energy to dormant microbial communities (Berney and Cook, 2010; Constant et al., 2010, 2011; Berney et al., 2014; Greening et al., 2015), as well as for reproduction.

It has been proposed that atmospheric chemosynthesis occurs increasingly within drier, more nutrient-starved soils (Ji et al., 2017; Bay et al., 2018), in part due to the exclusion of phototrophic microorganisms under moisture limitation (Warren-Rhodes et al., 2006; McKay, 2016). We found that the genetic capacity for atmospheric chemosynthesis was associated with increasingly drier, more nutrient-limited soils (Ji et al., 2017; Bay et al., 2018). Random forest and Pearson correlations revealed that *rbcL1E* and *hhyL*, relative to 16S rRNA, increased significantly across Antarctic and high Arctic soils that were increasingly limited in moisture and TC (Table 2 and Figure 5). Additionally, the relative abundance of *rbcL1E* also significantly increased in soils limited in NO_3_^−^ (Table 2 and Figure 5). Neither genetic determinant formed a significant positive correlation with bioavailable substances that are widely utilized by geothermal chemoautotrophic bacteria such as NO_2_^−^ and PO_4_^−^ (Figure 5; Engel, 2012). This lack of correlative data supports our current understanding that *rbcL1E* catalyzed primary production is not driven by geochemical energy sources. As a result, atmospheric chemosynthesis may occur within environments where soil nutrients are limited. Positive correlations were detected between *rbcL1E* and multiple trace oxides measured by X-Ray fluorescence elemental analysis (MnO, MgO, CaO, and Na_2_O; Table 2; Figure 5), suggesting a potential metabolic significance that requires further investigation.

It is recommended that additional studies are conducted to focus upon the isolation and characterization of trace gas chemosynthetic bacteria. Additionally, metagenomic and biochemical studies, including hydrogen oxidation and ^14^CO_2_ assimilation assays should be performed on a broader range of environments where atmospheric chemosynthesis is likely to occur. We suggest that sites should be targeted where organic carbon, water, and photoautotrophs are limited and the utilization of atmospheric gases by microbial communities is well documented (King, 2003; Lynch et al., 2012, 2014). This includes volcanic deposits as well as additional cold and hot deserts, such as the McMurdo Dry Valleys (Babalola et al., 2009; Van Goethem et al., 2016), Namib (van der Walt et al., 2016; Gunnigle et al., 2017), Thar (Rao et al., 2016), and Atacama (Lynch et al., 2014; Schulze-Makuch et al., 2018). Finally, this study highlights the genetic potential of microbial communities residing in cold oligotrophic deserts across the globe to conduct atmospheric chemosynthesis and their propensity for survival in regions with highly limited water and nutrient availability.

## Data Availability Statement

The datasets presented in this study can be found in online repositories described in the article. Original sequencing data is publicly available through NCBI under the accession number PRJNA645753. All other original contributions presented are included in the article/Supplementary Material.

## Author Contributions

BF determined the research objective with input from AR, MJ, and AT. AR conducted tag sequencing and qPCR. Soil parameter data for the Vestfold Hills was led by AT. Tag sequencing analysis was performed by AR and MJ. MJ and WK provided the Tibetan Plateau soil samples. AR, EZ, and MJ conducted multivariate data analysis, with input from BF. AR and BF wrote the manuscript with input from remaining authors. All authors contributed to the article and approved the submitted version.

### Conflict of Interest

The authors declare that the research was conducted in the absence of any commercial or financial relationships that could be construed as a potential conflict of interest.

## Footnotes

^1^http://dx.doi.org/10.4225/15/526F42ADA05B1

^2^https://data.bioplatforms.com/organization/about/australian-microbiome

^3^http://cran.r-project.org/

## References

[ref1] Albertsen M. Hugenholtz P. Skarshewski A. Nielsen K. Tyson G. Nielsen P. (2013). Genome sequences of rare, uncultured bacteria obtained by differential coverage binning of multiple metagenomes. Nat. Biotechnol. 31, 533–538. 10.1038/nbt.2579, PMID: 23707974

[ref2] Archer E. (2013). Estimate permutation p-values for importance metrics. R package version 1.5.2.

[ref3] Babalola O. O. Kirby B. M. Le Roes-Hill M. Cook A. E. Cary S. C. Burton S. G.. (2009). Phylogenetic analysis of actinobacterial populations associated with Antarctic dry valley mineral soils. Environ. Microbiol. 11, 566–576. 10.1111/j.1462-2920.2008.01809.x, PMID: 19278445

[ref4] Baugh L. R. Hill A. A. Brown E. L. Hunter C. P. (2001). Quantitative analysis of mRNA amplification by in vitro transcription. Nucleic Acids Res. 29:E29. 10.1093/nar/29.5.e29, PMID: 11222780 PMC29742

[ref5] Bay S. Ferrari B. Greening C. (2018). Life without water: how do bacteria generate biomass in desert ecosystems? Microbiol. Aust. 39, 28–32. 10.1071/MA18008

[ref6] Berney M. Cook G. M. (2010). Unique flexibility in energy metabolism allows mycobacteria to combat starvation and hypoxia. PLoS One 5:e8614. 10.1371/journal.pone.0008614, PMID: 20062806 PMC2799521

[ref7] Berney M. Greening C. Conrad R. Jacobs W. R. Cook G. M. (2014). An obligately aerobic soil bacterium activates fermentative hydrogen production to survive reductive stress during hypoxia. Proc. Natl. Acad. Sci. U. S. A. 111, 11479–11484. 10.1073/pnas.1407034111, PMID: 25049411 PMC4128101

[ref8] Bissett A. Fitzgerald A. Meintjes T. Mele P. M. Reith F. Dennis P. G.. (2016). Introducing BASE: the biomes of Australian soil environments soil microbial diversity database. Gigascience 5:21. 10.1186/s13742-016-0126-5, PMID: 27195106 PMC4870752

[ref9] Bottos E. Scarrow J. Archer S. McDonald I. Cary S. (2014). “Bacterial community structures of Antarctic soils” in Antarctic terrestrial microbiology. ed. Cowan D. (Berlin: Springer), 9–33.

[ref10] Cary S. McDonald I. Barrett J. Cowan D. (2010). On the rocks: the microbiology of Antarctic dry valley soils. Nat. Rev. Microbiol. 8, 129–138. 10.1038/nrmicro2281, PMID: 20075927

[ref11] Cherlet M. Hutchinson C. Reynolds J. Hill J., Sommer S., and von Maltitz G. (eds.) (2018). World atlas of desertification. 3rd Edn Luxembourg: Publication Office of the European Union.

[ref12] Clarke K. R. Warwick R. M. (2001). Changes in marine communities: An approach to statistical analysis and interpretation. Plymouth: PRIMER-E.

[ref13] Constant P. Chowdhury S. P. Hesse L. Pratscher J. Conrad R. (2011). Genome data mining and soil survey for the novel group 5 [NiFe]-Hydrogenase to explore the diversity and ecological importance of presumptive high-affinity H(2)-oxidizing bacteria. Appl. Environ. Microbiol. 77, 6027–6035. 10.1128/AEM.00673-11, PMID: 21742924 PMC3165403

[ref14] Constant P. Chowdhury S. P. Pratscher J. Conrad R. (2010). Streptomycetes contributing to atmospheric molecular hydrogen soil uptake are widespread and encode a putative high-affinity [NiFe]-hydrogenase. Environ. Microbiol. 12, 821–829. 10.1111/j.1462-2920.2009.02130.x, PMID: 20050876

[ref15] Constant P. Poissant L. Villemur R. (2008). Isolation of *Streptomyces* sp. PCB7, the first microorganism demonstrating high-affinity uptake of tropospheric H2. ISME J. 2, 1066–1076. 10.1038/ismej.2008.59, PMID: 18548118

[ref16] Cowan D. Makhalanyane T. Dennis P. Hopkins D. (2014). Microbial ecology and biogeochemistry of continental Antarctic soils. Front. Microbiol. 5:154. 10.3389/fmicb.2014.00154, PMID: 24782842 PMC3988359

[ref18] Edgar R. C. (2013). UPARSE: highly accurate OTU sequences from microbial amplicon reads. Nat. Methods 10, 996–998. 10.1038/nmeth.2604, PMID: 23955772

[ref19] Edgar R. C. Haas B. J. Clemente J. C. Quince C. Knight R. (2011). UCHIME improves sensitivity and speed of chimera detection. Bioinformatics 27, 2194–2200. 10.1093/bioinformatics/btr381, PMID: 21700674 PMC3150044

[ref20] Elster J. (2002). “Ecological classification of terrestrial algal communities in polar environments” in Geoecology of Antarctic ice-free coastal landscapes. eds. Beyer L. Bölter M. (Berlin, Heidelberg: Springer Berlin Heidelberg), 303–326.

[ref21] Engel A. S. (2012). “Chemoautotrophy” in Encyclopedia of caves. 2nd Edn eds. White W. B. Culver D. C. (Amsterdam: Academic Press), 125–134.

[ref22] Ferrari B. Bissett A. Snape I. van Dorst J. Palmer A. Ji M.. (2015). Geological connectivity drives microbial community structure and connectivity in polar, terrestrial ecosystems. Environ. Microbiol. 18, 1834–1849. 10.1111/1462-2920.13034, PMID: 26310523

[ref23] Friedmann E. I. (1982). Endolithic microorganisms in the Antartic cold desert. Science 215, 1045–1053. 10.1126/science.215.4536.1045, PMID: 17771821

[ref24] Gao Y. Wang W. Yao T. Lu N. Lu A. (2018). Hydrological network and classification of lakes on the third pole. J. Hydrol. 560, 582–594. 10.1016/j.jhydrol.2018.03.062

[ref25] Gasparon M. Ehrler K. Matschullat J. Melles M. (2007). Temporal and spatial variability of geochemical backgrounds in the Windmill Islands, East Antarctica: implications for climatic changes and human impacts. Appl. Geochem. 22, 888–905. 10.1016/j.apgeochem.2006.12.018

[ref26] Goodwin I. D. (1993). Holocene deglaciation, sea-level change, and the emergence of the Windmill Islands, Budd coast, Antarctica. Quat. Res. 40, 70–80. 10.1006/qres.1993.1057

[ref27] Goordial J. Davila A. Greer C. W. Cannam R. Diruggiero J. McKay C. P.. (2017). Comparative activity and functional ecology of permafrost soils and lithic niches in a hyper-arid polar desert. Environ. Microbiol. 19, 443–458. 10.1111/1462-2920.13353, PMID: 27129741

[ref28] Greening C. Berney M. Hards K. Cook G. M. Conrad R. (2014). A soil actinobacterium scavenges atmospheric H2 using two membrane-associated, oxygen-dependent [NiFe] hydrogenases. Proc. Natl. Acad. Sci. U. S. A. 111, 4257–4261. 10.1073/pnas.1320586111, PMID: 24591586 PMC3964045

[ref29] Greening C. Constant P. Hards K. Morales S. E. Oakeshott J. G. Russell R. J.. (2015). Atmospheric hydrogen scavenging: from enzymes to ecosystems. Appl. Environ. Microbiol. 81, 1190–1199. 10.1128/AEM.03364-14, PMID: 25501483 PMC4309691

[ref30] Grostern A. Alvarez-Cohen L. (2013). RubisCO-based CO2 fixation and C1 metabolism in the actinobacterium *Pseudonocardia* dioxanivorans CB1190. Environ. Microbiol. 15, 3040–3053. 10.1111/1462-2920.12144, PMID: 23663433

[ref31] Gunnigle E. Frossard A. Ramond J. -B. Guerrero L. Seely M. Cowan D. A. (2017). Diel-scale temporal dynamics recorded for bacterial groups in Namib desert soil. Sci. Rep. 7:40189. 10.1038/srep40189, PMID: 28071697 PMC5223211

[ref32] Islam Z. F. Cordero P. R. F. Feng J. Chen Y. -J. Bay S. K. Jirapanjawat T.. (2019). Two *Chloroflexi* classes independently evolved the ability to persist on atmospheric hydrogen and carbon monoxide. ISME J. 13, 1801–1813. 10.1038/s41396-019-0393-0, PMID: 30872805 PMC6776052

[ref33] Islam Z. F. Welsh C. Bayly K. Grinter R. Southam G. Gagen E. J.. (2020). A widely distributed hydrogenase oxidises atmospheric H_2_ during bacterial growth. ISME J. 10.1038/s41396-020-0713-4 [Epub ahead of print], PMID: 32647310 PMC7784904

[ref34] Jansson J. K. Taş N. (2014). The microbial ecology of permafrost. Nat. Rev. Microbiol. 12, 414–425. 10.1038/nrmicro3262, PMID: 24814065

[ref35] Ji M. Greening C. Vanwonterghem I. Carere C. R. Bay S. K. Steen J. A.. (2017). Atmospheric trace gases support primary production in Antarctic desert surface soil. Nature 552, 400–403. 10.1038/nature25014, PMID: 29211716

[ref36] Ji M. Kong W. Stegen J. Yue L. Wang F. Dong X.. (2020). Distinct assembly mechanisms underlie similar biogeographical patterns of rare and abundant bacteria in Tibetan plateau grassland soils. Environ. Microbiol. 22, 2261–2272. 10.1111/1462-2920.14993, PMID: 32216022

[ref37] Ji M. van Dorst J. Bissett A. Brown M. Palmer A. Snape I. (2015). Microbial diversity at Mitchell peninsula, eastern Antarctica: a potential biodiversity “hotspot”. Polar Biol. 39, 237–249. 10.1007/s00300-015-1776-y

[ref38] Jones C. M. Hallin S. (2019). Geospatial variation in co-occurrence networks of nitrifying microbial guilds. Mol. Ecol. 28, 293–306. 10.1111/mec.14893, PMID: 30307658 PMC6905385

[ref39] Kampstra P. (2008). Beanplot: a boxplot alternative for visual comparison of distributions. J. Stat. Softw. 28, 1–9. 10.18637/jss.v028.c0127774042

[ref40] Khdhiri M. Hesse L. Popa M. E. Quiza L. Lalonde I. Meredith L. K. (2015). Soil carbon content and relative abundance of high affinity H2-oxidizing bacteria predict atmospheric H2 soil uptake activity better than soil microbial community composition. Soil Biol. Biochem. 85, 1–9. 10.1016/j.soilbio.2015.02.030

[ref41] King G. M. (2003). Contributions of atmospheric CO and hydrogen uptake to microbial dynamics on recent Hawaiian volcanic deposits. Appl. Environ. Microbiol. 69, 4067–4075. 10.1128/AEM.69.7.4067-4075.2003, PMID: 12839783 PMC165208

[ref42] Kleinteich J. Hildebrand F. Bahram M. Voigt A. Y. Wood S. A. Jungblut A. D. (2017). Pole-to-pole connections: similarities between Arctic and Antarctic microbiomes and their vulnerability to environmental change. Front. Ecol. Evol. 5:137, 10.3389/fevo.2017.00137

[ref43] Lennon J. T. Jones S. E. (2011). Microbial seed banks: the ecological and evolutionary implications of dormancy. Nat. Rev. Microbiol. 9, 119–130. 10.1038/nrmicro2504, PMID: 21233850

[ref44] Lynch R. C. Darcy J. L. Kane N. C. Nemergut D. R. Schmidt S. K. (2014). Metagenomic evidence for metabolism of trace atmospheric gases by high-elevation desert *Actinobacteria*. Front. Microbiol. 5:698. 10.3389/fmicb.2014.00698, PMID: 25566214 PMC4269115

[ref45] Lynch R. C. King A. J. Farías M. E. Sowell P. Vitry C. Schmidt S. K. (2012). The potential for microbial life in the highest-elevation (>6000 m.a.s.l.) mineral soils of the Atacama region. J. Geophys. Res. Biogeosci. 117, 1–10. 10.1029/2012JG001961

[ref46] Maeda H. Fujimoto C. Haruki Y. Maeda T. Kokeguchi S. Petelin M.. (2003). Quantitative real-time PCR using TaqMan and SYBR green for *Actinobacillus actinomycetemcomitans*, *Porphyromonas gingivalis*, *Prevotella intermedia*, tetQgene and total bacteria. FEMS Immunol. Med. Microbiol. 39, 81–86. 10.1016/s0928-8244(03)00224-4, PMID: 14557000

[ref47] Mares M. A. History O. M. N. (1999). Encyclopedia of deserts. Norman, Oklahoma: University of Oklahoma Press.

[ref48] Margesin R. Miteva V. (2011). Diversity and ecology of psychrophilic microorganisms. Res. Microbiol. 162, 346–361. 10.1016/j.resmic.2010.12.004, PMID: 21187146

[ref49] McKay C. P. (2016). Water sources for *Cyanobacteria* below desert rocks in the Negev desert determined by conductivity. Glob. Ecol. Conserv. 6, 145–151. 10.1016/j.gecco.2016.02.010

[ref50] Meredith L. K. Rao D. Bosak T. Klepac-Ceraj V. Tada K. R. Hansel C. M.. (2014). Consumption of atmospheric hydrogen during the life cycle of soil-dwelling *Actinobacteria*. Environ. Microbiol. Rep. 6, 226–238. 10.1111/1758-2229.12116, PMID: 24983527

[ref51] Morita R. Y. (1999). Is H(2) the universal energy source for long-term survival? Microb. Ecol. 38, 307–320. 10.1007/s002489901002, PMID: 10758178

[ref53] Namsaraev Z. Mano M. J. Fernandez R. Wilmotte A. (2010). Biogeography of terrestrial cyanobacteria from Antarctic ice-free areas. Ann. Glaciol. 51, 171–177. 10.3189/172756411795931930

[ref55] Oksanen J. Blanchet F. G. Friendly M. Kindt R. Legendre P. McGlinn D. (2015). Vegan: Community ecology packageR package version 2.5–2. Available at: https://CRAN.R-project.org/package=vegan (Accessed November 16, 2018).

[ref56] Park S. W. Hwang E. H. Jang H. S. Lee J. H. Kang B. S. Oh J. I.. (2009). Presence of duplicate genes encoding a phylogenetically new subgroup of form I ribulose 1,5-bisphosphate carboxylase/oxygenase in *Mycobacterium* sp. strain JC1 DSM 3803. Res. Microbiol. 160, 159–165. 10.1016/j.resmic.2008.12.002, PMID: 19135529

[ref57] Pearce D. A. (2012). “Extremophiles in Antarctica: life at low temperatures” in Adaption of microbial life to environmental extremes. eds. Stan-Lotter H. Fendrihan S. (New York: Springer-Verlag/Wien).

[ref58] Piche-Choquette S. Constant P. (2019). Molecular hydrogen, a neglected key driver of soil biogeochemical processes. Appl. Environ. Microbiol. 85:e02418. 10.1128/AEM.02418-18, PMID: 30658976 PMC6414374

[ref59] Piché-Choquette S. Tremblay J. Tringe S. G. Constant P. (2016). H2-saturation of high affinity H2-oxidizing bacteria alters the ecological niche of soil microorganisms unevenly among taxonomic groups. PeerJ 4:e1782. 10.7717/peerj.1782, PMID: 26989620 PMC4793312

[ref60] Pointing S. B. Chan Y. Lacap D. C. Lau M. C. Y. Jurgens J. A. Farrell R. L. (2009). Highly specialized microbial diversity in hyper-arid polar desert. Proc. Natl. Acad. Sci. U. S. A. 106, 19964–19969. 10.1073/pnas.0908274106, PMID: 19850879 PMC2765924

[ref61] Pudasaini S. Wilson J. Ji M. van Dorst J. Snape I. Palmer A. S.. (2017). Microbial diversity of Browning peninsula, Eastern Antarctica revealed using molecular and cultivation methods. Front. Microbiol. 8:591. 10.3389/fmicb.2017.00591, PMID: 28439263 PMC5383709

[ref63] Quast C. Pruesse E. Yilmaz P. Gerken J. Schweer T. Yarza P.. (2013). The SILVA ribosomal RNA gene database project: improved data processing and web-based tools. Nucleic Acids Res. 41, D590–D596. 10.1093/nar/gks1219, PMID: 23193283 PMC3531112

[ref64] R Core Team (2018). R: A Language and environment for statistical computing. R Foundation for Statistical Computing, Vienna. Available at: https://www.R-project.org (Accessed November 16, 2018).

[ref65] Rao S. Chan Y. Bugler-Lacap D. C. Bhatnagar A. Bhatnagar M. Pointing S. B. (2016). Microbial diversity in soil, sand dune and rock substrates of the Thar Monsoon desert, India. Indian J. Microbiol. 56, 35–45. 10.1007/s12088-015-0549-1, PMID: 26843695 PMC4729749

[ref66] Rayment R. E. Lyons D. J. (2011). Soil chemical methods-Australasia. Collingwood, VIC: CSIRO Publishing.

[ref67] RStudio and Inc (2013). Shiny: Web application framework for R. R package version 0.5.0.

[ref68] Sato Y. Nishihara H. Yoshida M. Watanabe M. Rondal J. D. Ohta H. (2004). Occurrence of hydrogen-oxidizing *Ralstonia* species as primary microorganisms in the Mt. Pinatubo volcanic mudflow deposits. Soil Sci. Plant Nutr. 50, 855–861. 10.1080/00380768.2004.10408546

[ref70] Schulze-Makuch D. Wagner D. Kounaves S. P. Mangelsdorf K. Devine K. G. de Vera J. -P.. (2018). Transitory microbial habitat in the hyperarid Atacama desert. Proc. Natl. Acad. Sci. U. S. A. 115, 2670–2675. 10.1073/pnas.1714341115, PMID: 29483268 PMC5856521

[ref71] Siciliano S. D. Palmer A. S. Winsley T. Lamb E. Bissett A. Brown M. V. (2014). Soil fertility is associated with fungal and bacterial richness, whereas pH is associated with community composition in polar soil microbial communities. Soil Biol. Biochem. 78, 10–20. 10.1016/j.soilbio.2014.07.005

[ref72] Smith-Downey N. V. Randerson J. T. Eiler J. M. (2008). Molecular hydrogen uptake by soils in forest, desert, and marsh ecosystems in California. J. Geophys. Res. Biogeosci. 113, 1–11. 10.1029/2008JG000701

[ref73] Tebo B. Davis R. Anitori R. Connell L. Schiffman P. Staudigel H. (2015). Microbial communities in dark oligotrophic volcanic ice cave ecosystems of Mt. Erebus, Antarctica. Front. Microbiol. 6:179. 10.3389/fmicb.2015.00179, PMID: 25814983 PMC4356161

[ref74] Tindall B. J. (2004). Prokaryotic diversity in the Antarctic: the tip of the iceberg. Microb. Ecol. 47, 271–283. 10.1007/s00248-003-1050-7, PMID: 15054676

[ref75] Torbjørn R. Tomáš F. Ben N. Christopher Q. Frédéric M. (2016). VSEARCH: a versatile open source tool for metagenomics. PeerJ 4:e2584. 10.7717/peerj.2584, PMID: 27781170 PMC5075697

[ref76] Torondel B. Ensink J. H. J. Gundogdu O. Ijaz U. Z. Parkhill J. Abdelahi F.. (2016). Assessment of the influence of intrinsic environmental and geographical factors on the bacterial ecology of pit latrines. Microb. Biotechnol. 9, 209–223. 10.1111/1751-7915.12334, PMID: 26875588 PMC4767293

[ref77] Tytgat B. Verleyen E. Obbels D. Peeters K. De Wever A. D’hondt S.. (2014). Bacterial diversity assessment in Antarctic terrestrial and aquatic microbial mats: a comparison between bidirectional pyrosequencing and cultivation. PLoS One 9:e97564. 10.1371/journal.pone.0097564, PMID: 24887330 PMC4041716

[ref78] van der Walt A. J. Johnson R. M. Cowan D. A. Seely M. Ramond J. -B. (2016). Unique microbial phylotypes in Namib desert dune and gravel plain fairy circle soils. Appl. Environ. Microbiol. 82, 4592–4601. 10.1128/AEM.00844-16, PMID: 27208111 PMC4984285

[ref79] van Dorst J. Bissett A. Palmer A. S. Brown M. Snape I. Stark J. S.. (2014b). Community fingerprinting in a sequencing world. FEMS Microbiol. Ecol. 89, 316–330. 10.1111/1574-6941.12308, PMID: 24580036

[ref80] van Dorst J. Siciliano S. Winsley T. Snape I. Ferrari B. (2014a). Bacterial targets as potential indicators of diesel fuel toxicity in Subantarctic soils. Appl. Environ. Microbiol. 80, 4021–4033. 10.1128/aem.03939-13, PMID: 24771028 PMC4054227

[ref81] Van Goethem M. W. Makhalanyane T. P. Valverde A. Cary S. C. Cowan D. A. (2016). Characterization of bacterial communities in lithobionts and soil niches from Victoria valley. FEMS Microbiol. Ecol. 92:fiw051. 10.1093/femsec/fiw051, PMID: 26946500

[ref82] Villalva C. Touriol C. Seurat P. Trempat P. Delsol G. Brousset P. (2001). Increased yield of PCR products by addition of T4 gene 32 protein to the SMART PCR cDNA synthesis system. Biotechniques 31, 81–86. 10.2144/01311st04, PMID: 11464524

[ref83] Walker J. J. Pace N. R. (2007). Endolithic microbial ecosystems. Annu. Rev. Microbiol. 61, 331–347. 10.1146/annurev.micro.61.080706.093302, PMID: 17506683

[ref84] Warren-Rhodes K. A. Rhodes K. L. Pointing S. B. Ewing S. A. Lacap D. C. Gómez-Silva B.. (2006). Hypolithic cyanobacteria, dry limit of photosynthesis, and microbial ecology in the Hyperarid Atacama desert. Microb. Ecol. 52, 389–398. 10.1007/s00248-006-9055-7, PMID: 16865610

[ref85] Wei T. Simko V. Levy M. Xie Y. Jin Y. Zemla J. (2017). Package ‘corrplot’. Statistician 56:e24.

[ref86] Wickham H. (2016). ggplot2: Elegant graphics for data analysis. Springer International Publishing.

[ref87] Wierzchos J. de Los Rios A. Ascaso C. (2012). Microorganisms in desert rocks: the edge of life on earth. Int. Microbiol. 15, 172–182. 10.2436/20.1501.01.170, PMID: 23844476

[ref88] Wynn-Williams D. D. (1990). “Ecological aspects of Antarctic microbiology” in Advances in microbial ecology. ed. Marshall K. C. (Boston, MA: Springer US), 71–146.

[ref89] Ye J. Coulouris G. Zaretskaya I. Cutcutache I. Rozen S. Madden T. (2012). Primer-BLAST: a tool to design target-specific primers for polymerase chain reaction. BMC Bioinformatics 13:134. 10.1186/1471-2105-13-134, PMID: 22708584 PMC3412702

[ref90] Yergeau E. Bokhorst S. Huiskes A. Boschker H. Aerts R. Kowalchuk G. (2006). Size and structure of bacterial, fungal and nematode communities along an Antarctic environmental gradient. FEMS Microbiol. Ecol. 59, 436–451. 10.1111/j.1574-6941.2006.00200.x, PMID: 16978243

[ref92] Zhang E. Thibaut L. M. Terauds A. Wong S. van Dorst J. Tanaka M. M.. (2020). Lifting the veil on arid-to-hyperarid Antarctic soil microbiomes: a tale of two oases. Microbiome 8:37. 10.1186/s40168-020-00809-w, PMID: 32178729 PMC7076931

