# Supplementary material for: Soil Microbiomes With the Genetic Capacity for Atmospheric Chemosynthesis Are Widespread Across the Poles and Are Associated With Moisture, Carbon, and Nitrogen Limitation
Source: Front Microbiol. 2020 Aug 12;11:1936. doi: 10.3389/fmicb.2020.01936 (PMC7437527; doi:10.3389/fmicb.2020.01936)

**Supplementary Information 1.** Geographical, physical and chemical parameters of the Vestfold Hill soil samples

|  | **AF** | | **HV** | | **OW** | | **RL** | | **TR** | |
| --- | --- | --- | --- | --- | --- | --- | --- | --- | --- | --- |
|  | **Mean** | **SD** | **Mean** | **SD** | **Mean** | **SD** | **Mean** | **SD** | **Mean** | **SD** |
| **Elevation (m)** | 2.996 | 1.126 | 11.712 | 2.783 | 18.895 | 0.658 | 12.146 | 2.918 | 27.894 | 2.885 |
| **Aspect (deg)** | 137.511 | 129.780 | 145.097 | 147.428 | 201.283 | 93.085 | 163.982 | 51.951 | 205.018 | 118.285 |
| **Mud (%)** | 5.755 | 3.677 | 30.567 | 8.707 | 24.632 | 16.065 | 26.329 | 11.446 | N/A | N/A |
| **Sand (%)** | 88.010 | 4.445 | 58.897 | 7.284 | 67.321 | 14.344 | 62.100 | 12.899 | N/A | N/A |
| **Dry Matter Fraction (%)** | 0.984 | 0.011 | 0.919 | 0.016 | 0.923 | 0.045 | 0.925 | 0.038 | 0.963 | 0.033 |
| **TN (ppm)** | 111.889 | 49.154 | 165.556 | 23.511 | 340.556 | 252.642 | 178.889 | 55.777 | N/A | N/A |
| **TP (ppm)** | 956.667 | 296.648 | 741.111 | 79.127 | 675.556 | 121.769 | 1744.444 | 415.665 | N/A | N/A |
| **TC (ppm)** | 1034.389 | 427.084 | 1742.556 | 303.180 | 4494.111 | 2694.401 | 1542.667 | 392.302 | 776.126 | 575.660 |
| **SiO2 (%)** | 63.402 | 1.417 | 61.634 | 1.110 | 60.684 | 1.427 | 57.479 | 1.219 | 59.563 | 1.612 |
| **TiO2 (%)** | 0.784 | 0.103 | 0.848 | 0.031 | 0.840 | 0.105 | 1.061 | 0.120 | 0.960 | 0.114 |
| **Al2O3 (%)** | 13.882 | 0.355 | 13.331 | 0.185 | 12.997 | 0.635 | 14.747 | 0.585 | 14.134 | 0.501 |
| **Fe2O3 (%)** | 7.630 | 0.796 | 9.232 | 0.488 | 9.375 | 1.517 | 9.834 | 0.476 | 9.488 | 0.848 |
| **MnO (%)** | 0.116 | 0.010 | 0.131 | 0.011 | 0.138 | 0.027 | 0.125 | 0.010 | 0.121 | 0.014 |
| **MgO (%)** | 3.989 | 0.268 | 4.701 | 0.235 | 4.769 | 0.321 | 4.574 | 0.524 | 4.528 | 0.660 |
| **CaO (%)** | 5.217 | 0.141 | 5.063 | 0.395 | 5.156 | 0.401 | 5.336 | 0.682 | 5.217 | 0.402 |
| **Na2O (%)** | 3.475 | 0.170 | 2.932 | 0.176 | 3.230 | 0.567 | 3.784 | 0.193 | 3.799 | 0.640 |
| **K2O (%)** | 1.477 | 0.119 | 1.631 | 0.158 | 1.531 | 0.146 | 2.351 | 0.312 | 1.746 | 0.258 |
| **P2O5 (%)** | 0.198 | 0.041 | 0.194 | 0.007 | 0.179 | 0.022 | 0.404 | 0.097 | 0.256 | 0.048 |
| **Conductivity (uS/cm)** | 1668.222 | 1559.440 | 1675.667 | 1590.912 | 7119.300 | 7129.969 | 2923.333 | 2374.121 | 2049.689 | 2916.998 |
| **pH** | 8.022 | 0.708 | 8.911 | 0.857 | 8.600 | 0.737 | 7.911 | 0.852 | 8.992 | 0.564 |
| **Cl (ppm)** | 1071.213 | 1967.535 | 1015.609 | 2347.431 | 1702.767 | 3726.202 | 2233.651 | 3243.430 | 1178.000 | 1205.112 |
| **NO2 (ppm)** | 0.287 | 0.271 | 0.395 | 0.291 | 0.168 | 0.053 | 0.281 | 0.260 | 0.411 | 0.333 |
| **Br (ppm)** | 1.356 | 1.658 | 0.294 | 0.433 | 6.896 | 8.789 | 2.625 | 1.577 | N/A | N/A |
| **NO3 (ppm)** | 5.535 | 5.002 | 2.870 | 4.998 | 8.712 | 14.693 | 17.139 | 15.760 | 3.922 | 4.449 |
| **PO4 (ppm)** | 3.016 | 5.799 | 3.813 | 3.563 | 0.791 | 1.011 | 4.362 | 3.733 | 2.067 | 1.809 |
| **SO4 (ppm)** | 704.927 | 808.991 | 333.473 | 305.660 | 1547.279 | 3075.893 | 284.707 | 360.709 | 1059.111 | 2238.786 |

**Supplementary Information 2.** Geographical, physical and chemical parameters of the Windmill Island soil samples

|  | **BP** | | **CS** | | **HI** | | **MP** | | **RR** | |
| --- | --- | --- | --- | --- | --- | --- | --- | --- | --- | --- |
|  | **Mean** | **SD** | **Mean** | **SD** | **Mean** | **SD** | **Mean** | **SD** | **Mean** | **SD** |
| **Elevation (m)** | 41.238 | 1.479 | 35.557 | 0.105 | 30.866 | 4.486 | 31.647 | 17.018 | 35.568 | 8.993 |
| **Aspect (deg)** | 170.399 | 94.789 | 167.411 | 174.461 | 130.400 | 153.251 | 217.094 | 147.787 | 118.812 | 149.603 |
| **Mud (%)** | 4.161 | 1.032 | 13.294 | 2.900 | 3.053 | 1.463 | 1.607 | 2.296 | 6.377 | 3.042 |
| **Sand (%)** | 70.231 | 6.108 | 64.536 | 3.520 | 69.310 | 5.948 | 64.366 | 14.241 | 69.271 | 6.232 |
| **Dry Matter Fraction (%)** | 0.892 | 0.034 | 0.834 | 0.068 | 0.963 | 0.016 | 0.972 | 0.010 | 0.905 | 0.049 |
| **TN (ppm)** | 218.889 | 87.528 | 457.143 | 273.966 | 155.556 | 104.057 | 372.222 | 215.045 | 954.444 | 692.858 |
| **TP (ppm)** | 818.889 | 153.740 | 1862.857 | 823.199 | 1096.667 | 307.327 | 471.111 | 145.983 | 1588.889 | 428.499 |
| **TC (ppm)** | 2222.222 | 611.919 | 6288.889 | 5072.338 | 777.778 | 432.371 | 2910.444 | 1449.648 | 10833.333 | 8972.597 |
| **SiO2 (%)** | 67.858 | 1.128 | 69.315 | 2.765 | 62.482 | 2.643 | 66.166 | 6.078 | 63.874 | 3.062 |
| **TiO2 (%)** | 0.976 | 0.128 | 0.404 | 0.089 | 1.088 | 0.215 | 0.645 | 0.369 | 0.948 | 0.077 |
| **Al2O3 (%)** | 13.447 | 0.194 | 13.546 | 0.530 | 13.996 | 0.368 | 14.818 | 1.844 | 13.755 | 0.395 |
| **Fe2O3 (%)** | 6.016 | 0.778 | 4.009 | 0.594 | 7.787 | 1.692 | 6.921 | 4.224 | 6.232 | 0.501 |
| **MnO (%)** | 0.114 | 0.013 | 0.125 | 0.017 | 0.155 | 0.026 | 0.214 | 0.151 | 0.131 | 0.011 |
| **MgO (%)** | 1.399 | 0.115 | 1.316 | 0.320 | 3.186 | 0.827 | 1.972 | 1.042 | 1.993 | 0.916 |
| **CaO (%)** | 3.350 | 0.080 | 2.750 | 0.534 | 5.405 | 0.784 | 2.312 | 0.598 | 4.047 | 1.041 |
| **Na2O (%)** | 2.798 | 0.079 | 3.097 | 0.145 | 2.936 | 0.128 | 2.769 | 0.606 | 2.837 | 0.110 |
| **K2O (%)** | 3.152 | 0.188 | 3.005 | 0.113 | 2.726 | 0.355 | 3.108 | 0.651 | 3.071 | 0.204 |
| **P2O5 (%)** | 0.202 | 0.018 | 0.552 | 0.273 | 0.277 | 0.080 | 0.153 | 0.021 | 0.386 | 0.096 |
| **Conductivity (uS/cm)** | 36.222 | 19.059 | 65.800 | 39.398 | 266.678 | 286.471 | 48.522 | 15.450 | 25.311 | 15.371 |
| **pH** | 6.584 | 0.107 | 6.648 | 0.780 | 6.581 | 0.173 | 5.351 | 0.307 | 5.236 | 0.273 |
| **Cl (ppm)** | 36.278 | 23.933 | 49.469 | 51.218 | 182.758 | 93.955 | 51.248 | 25.971 | 6.460 | 2.960 |
| **NO2 (ppm)** | 0.136 | 0.182 | 0.445 | 0.979 | 0.075 | 0.000 | 0.075 | 0.000 | 0.186 | 0.333 |
| **Br (ppm)** | 0.075 | 0.000 | 0.075 | 0.000 | 0.280 | 0.493 | 0.075 | 0.000 | 0.155 | 0.180 |
| **NO3 (ppm)** | 2.382 | 1.027 | 2.397 | 1.800 | 1.233 | 0.970 | 0.458 | 0.197 | 0.868 | 0.191 |
| **PO4 (ppm)** | 1.601 | 0.469 | 14.380 | 8.132 | 4.169 | 2.545 | 0.690 | 0.885 | 5.043 | 3.104 |
| **SO4 (ppm)** | 8.285 | 3.185 | 34.490 | 32.395 | 37.673 | 18.263 | 12.881 | 6.955 | 7.824 | 6.394 |

**Supplementary Information 3.** Geographical, physical and chemical parameters of the high Arctic soil samples

|  | **AFH** | | **SS** | | **SV** | |
| --- | --- | --- | --- | --- | --- | --- |
|  | **Mean** | **SD** | **Mean** | **SD** | **Mean** | **SD** |
| **Elevation (m)** | 509.556 | 11.092 | 84.000 | 30.336 | 5.750 | 1.035 |
| **Aspect (deg)** | 9.300 | 4.426 | 28.789 | 11.303 | 198.613 | 67.695 |
| **Mud (%)** | 2.431 | 0.197 | 2.489 | 0.171 | 2.736 | 0.146 |
| **Sand (%)** | 51.990 | 10.888 | 44.323 | 7.986 | 33.116 | 3.809 |
| **Dry Matter Fraction (%)** | 0.923 | 0.022 | 0.813 | 0.061 | 0.720 | 0.080 |
| **TN (ppm)** | 484.444 | 176.147 | 1477.778 | 460.890 | 2425.000 | 1081.996 |
| **TP (ppm)** | 207.778 | 35.629 | 725.556 | 76.503 | 558.750 | 120.290 |
| **TC (ppm)** | 32188.889 | 25368.605 | 30267.778 | 8879.061 | 28935.000 | 16356.521 |
| **SiO2 (%)** | 52.323 | 15.343 | 60.134 | 3.614 | 66.640 | 3.067 |
| **TiO2 (%)** | 0.472 | 0.144 | 0.891 | 0.057 | 0.671 | 0.024 |
| **Al2O3 (%)** | 11.890 | 3.297 | 16.225 | 1.087 | 12.704 | 0.317 |
| **Fe2O3 (%)** | 5.304 | 0.972 | 6.776 | 0.500 | 5.195 | 0.239 |
| **MnO (%)** | 0.109 | 0.023 | 0.060 | 0.007 | 0.041 | 0.014 |
| **MgO (%)** | 5.271 | 3.880 | 1.411 | 0.223 | 0.949 | 0.051 |
| **CaO (%)** | 8.506 | 6.471 | 0.575 | 0.043 | 0.564 | 0.094 |
| **Na2O (%)** | 1.921 | 0.707 | 2.033 | 0.330 | 1.593 | 0.101 |
| **K2O (%)** | 1.995 | 0.392 | 2.741 | 0.227 | 2.582 | 0.048 |
| **P2O5 (%)** | 0.090 | 0.013 | 0.210 | 0.012 | 0.151 | 0.018 |
| **Conductivity (uS/cm)** | 59.714 | 35.720 | 36.904 | 12.257 | 77.775 | 20.259 |
| **pH** | 7.807 | 0.622 | 6.213 | 0.357 | 5.688 | 0.201 |
| **Cl (ppm)** | 10.151 | 8.974 | 7.897 | 3.907 | 12.824 | 4.080 |
| **NO2 (ppm)** | 0.434 | 0.427 | 0.075 | 0.000 | 0.074 | 0.003 |
| **Br (ppm)** | 0.511 | 0.660 | 0.075 | 0.000 | 0.336 | 0.402 |
| **NO3 (ppm)** | 2.898 | 2.043 | 1.959 | 1.484 | 2.509 | 1.035 |
| **PO4 (ppm)** | 0.353 | 0.432 | 0.533 | 0.593 | 0.604 | 0.993 |
| **SO4 (ppm)** | 1.579 | 0.270 | 11.712 | 4.030 | 48.341 | 17.438 |

**Supplementary Information 4.** Specificity of the qPCR primer set targeting RuBisCO type IE in polar desert soils

|  |  | **RuBisCO type (%)** | | | |  |
| --- | --- | --- | --- | --- | --- | --- |
| **Site** | **Total Reads** | **IE** | **IC** | **IA** | **IB** | **Non-RuBisCO (%)** |
| **Robinson Ridge** | 49211 | 86.5 | 12.0 | 0.0 | 0.8 | 0.7 |
| **Herring Island** | 72230 | 85.3 | 11.7 | 2.0 | 0.0 | 1.1 |
| **Casey Station** | 57955 | 71.8 | 24.8 | 0.1 | 0.1 | 3.3 |
| **Mitchell Peninsula** | 75328 | 86.9 | 7.2 | 0.0 | 0.0 | 5.8 |
| **Browning Peninsula** | 77511 | 62.4 | 34.6 | 1.6 | 0.0 | 1.3 |
| **Old Wallow** | 56115 | 61.9 | 31.3 | 3.6 | 0.0 | 3.3 |
| **Heidemann Valley** | 63646 | 74.0 | 18.2 | 5.8 | 0.0 | 1.9 |
| **Rookery Lake** | 68893 | 76.3 | 13.6 | 7.5 | 0.0 | 2.6 |
| **Adams Flat** | 67887 | 72.3 | 17.7 | 8.5 | 0.0 | 1.5 |
| **The Ridge** | 59958 | 77.7 | 17.0 | 2.6 | 0.0 | 2.7 |
| **Alexandra Fjord Highlands** | 73953 | 61.5 | 23.1 | 3.8 | 0.0 | 11.6 |
| **Tibetan Plateau** | 38168 | 69.2 | 12.2 | 12.6 | 0.0 | 6.0 |
| **Average** | 63405 | 73.8 | 18.6 | 4.0 | 0.1 | 3.5 |

**Supplementary Information 5**. Soil bacterial community diversity across all 14 cold desert sites at phylum level, displayed as bar charts.


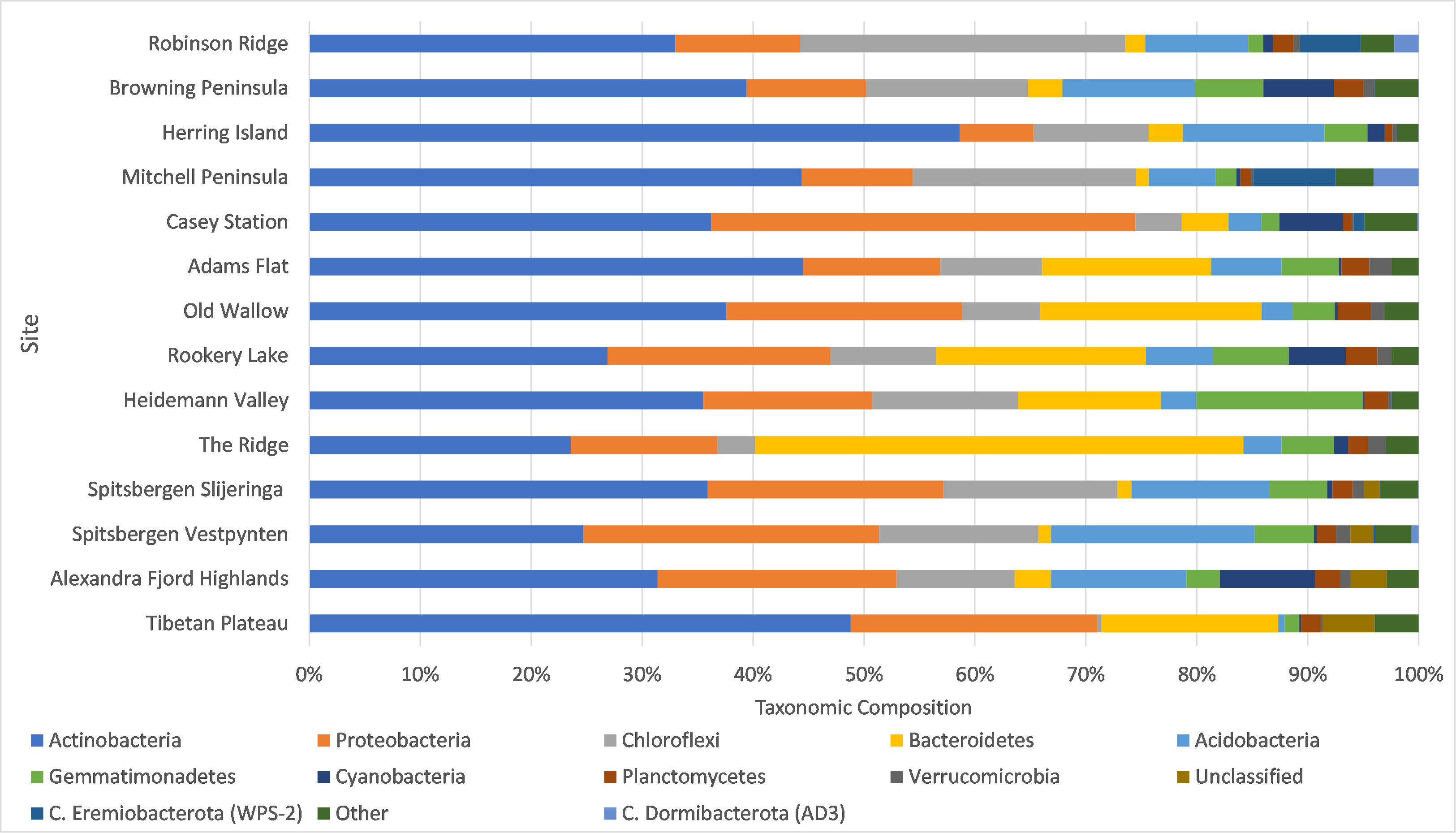

Supplement: Supplementary file 1 [file Data_Sheet_1.DOCX]
